# Development of a reverse transcription recombinase-aided amplification assay for detection of Getah virus

**DOI:** 10.1038/s41598-021-99734-7

**Published:** 2021-10-08

**Authors:** Mincai Nie, Huidan Deng, Yuancheng Zhou, Xiangang Sun, Yao Huang, Ling Zhu, Zhiwen Xu

**Affiliations:** 1 College of Veterinary Medicine, Sichuan Agricultural University, Chengdu, 611130 China; 2 College of Veterinary Medicine Sichuan Key Laboratory of Animal Epidemic Disease and Human Health, Sichuan Agricultural University, Chengdu, 611130 China; 3 Livestock and Poultry Biological Products Key Laboratory of Sichuan Province, Sichuan Animal Science Academy, Chengdu, 610066 China

**Keywords:** Biological techniques, Molecular biology

## Abstract

GETV, an arbo-borne zoonotic virus of the genus Alphavirus, which causes diarrhea and reproduction disorders in swine, lead to serious economic losses to the swine industry in China. At present, the existing methods for GETV detection are time-consuming and low sensitivity, so, a rapid, accurate and sensitive GETV detection method is urgently needed. In this study, a fluorescent reverse transcription recombinase-assisted amplification method (RT-RAA) was successfully established for the rapid detection of GETV. The sensitivity of this method to GETV was 8 copies/reaction and 20 TCID_50_/reaction. No cross-reaction with other viruses. A total of 118 samples were prepared for GETV detection using fluorescent RT-RAA and SYBR Green I RT-qPCR, the coincidence rate of the two methods was 100%. The results suggest that the RT-RAA method is rapid, sensitive and specific for GETV detection and can be applied in the clinical.

## Introduction

Getah virus (GETV), a zoonotic virus belonging to the genus alphavirus, is a plus-strand RNA virus^1^. Since GETV was first isolated in Malaysia in 1955^2^, GETV has been found in Australia, China, Japan, Mongolia, Russia and many other countries^3^. It mainly causes fever, diarrhea and reproductive disorders in swines, lead to rash, edema and fever in horses^4–6^.

GETV can infect a wide range of hosts, including pigs, horses, mammals such as cattle and blue foxes^7–10^. Chickens, ducks and humans may also be its hosts. Li et al.^3^ used plaque reduction neutralization test and found that chickens (2.17%, 1/46) and ducks (5.56%, 1/18) also contained GETV neutralization antibodies, suggesting that chickens and ducks may also be hosts of GETV. In the serological survey of the human, GETV specific antibodies were detected in Guangdong, Hainan, Yunnan and Hebei^11–14^, and the positive rate of GETV IgG in Hebei was as high as 16.3% (49/300)^15^. The positive rate of GETV IgM antibody detected in the sera of febrile patients in summer was 0.63% (2/317)^16^. Although GETV has not been proven to cause human diseases, the presence of GETV-specific antibodies in fetid patients or healthy people in endemic areas suggests that humans are susceptible to the virus and may be a potential pathogen of human diseases.

GETV was first found to infect and cause disease in swine in Japan in 1985, and subsequent outbreaks of porcine GETV have been reported in Asian Countries. In China, the first outbreak of GETV in swines was found in Hunan province in 2017, resulting in the death of 200 piglets after birth and abortion of 150 pregnant sows^17^. After that, the threat of GETV to the pig industry is becoming more and more serious. In the detection of GETV pathogens from 231 swine farms in Henan, Hebei, Shanxi and Anhui provinces, the positive rate of samples was 4.62%, and the positive rate of swine farms was 13.9%^18^. GETV has become a serious threat to China's swine industry, and the rapid diagnosis of GETV is particularly important for the prevention and control of GETV.

Recombinase aided amplification (RAA) is a new nucleic acid detection technology in recent years. By adding Recombinase and binding protein, RAA can quickly detect the nucleic acid amplification at 39 °C, and the result can be determined within 15–30 min. Fluorescent RAA is the combination of probe qPCR and RAA, which retains the high specificity and sensitivity of probe qPCR and combines the advantages of rapid and accurate RAA. Therefore, fluorescent RAA has the advantages of high sensitivity, strong specificity, high accuracy and short detection time, etc., and has been widely used in the clinical detection of human and animal pathogens, such as novel coronavirus, tick-borne encephalitis virus, African swine fever virus and dengue virus^19–22^.

Whereas, there are no reports of using RAA to detect GETV. In this study, we aimed to establish a fast and efficient fluorescence RT-RAA method for detecting GETV, and conducted a preliminary evaluation of this method.

## Results

### Results of primer selection

**Figure 1:**
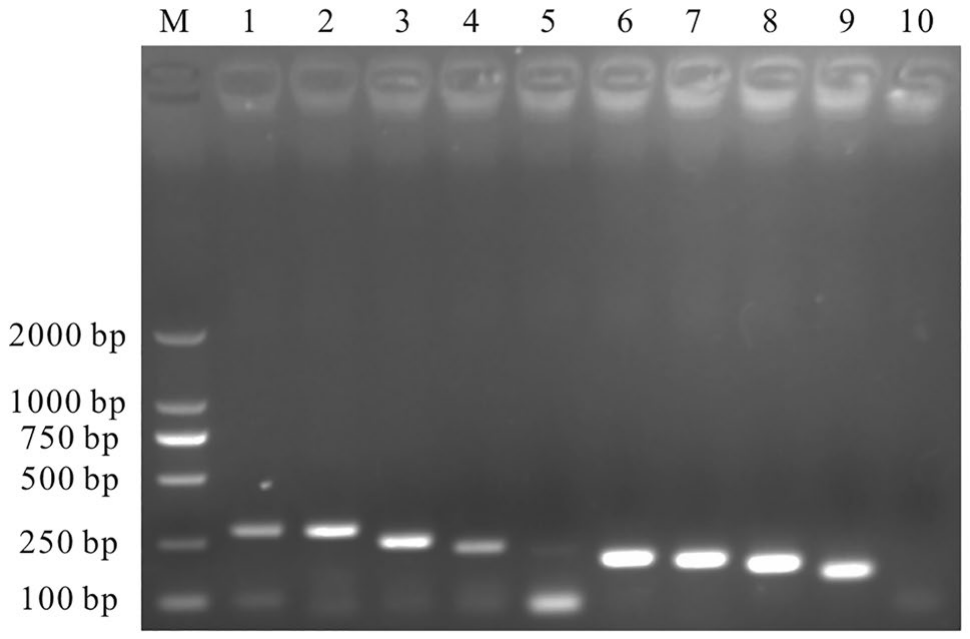
RT-PCR results. M: DL2000 Marker; 1–9: 9 pairs of GETV primers; 10: negative control.

**Figure 2:**
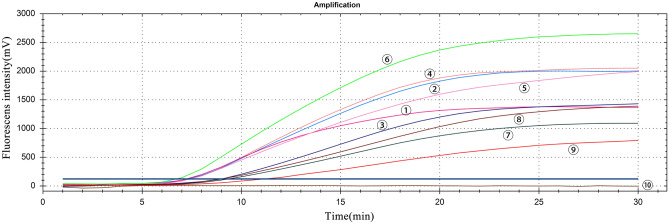
Results of primer selection. ①–⑨: 9 pairs of primers; ⑩: negative control. The fluorescens intensity of the curve: ⑥: 2637 mV, ④: 2044 mV, ②: 2000 mV, ⑤: 1937 mV, ③: 1411 mV, ①: 1373 mV, ⑧: 1360 mV, ⑦: 1085 mV, ⑨ 762 mV.

All the 9 pairs of primers were able to amplify GETV by RT-PCR (Fig. 1), and the amplified products were identified as GETV sequences. Among the 9 pairs of primers, the reaction system of RT-RAA-F2 and RT-RAA-R3 had the highest fluorescence value and an early peak (Fig. 2). Thus, the combination of RT-RAA-F2 and RT-RAA-R3 was used as primer for RT-RAA in subsequent experiments.

### Analytical sensitivity and specificity of the real-time RT-RAA assay

**Figure 3:**
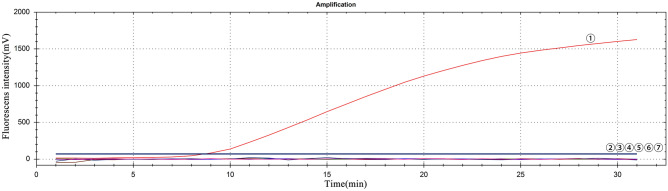
Specificity of the RT-RAA assay for GETV. ①–⑥: GETV, PRRSV, JEV, CSFV, APPV and SVV virus RNA respectively; ⑦: negative control.

**Figure 4:**
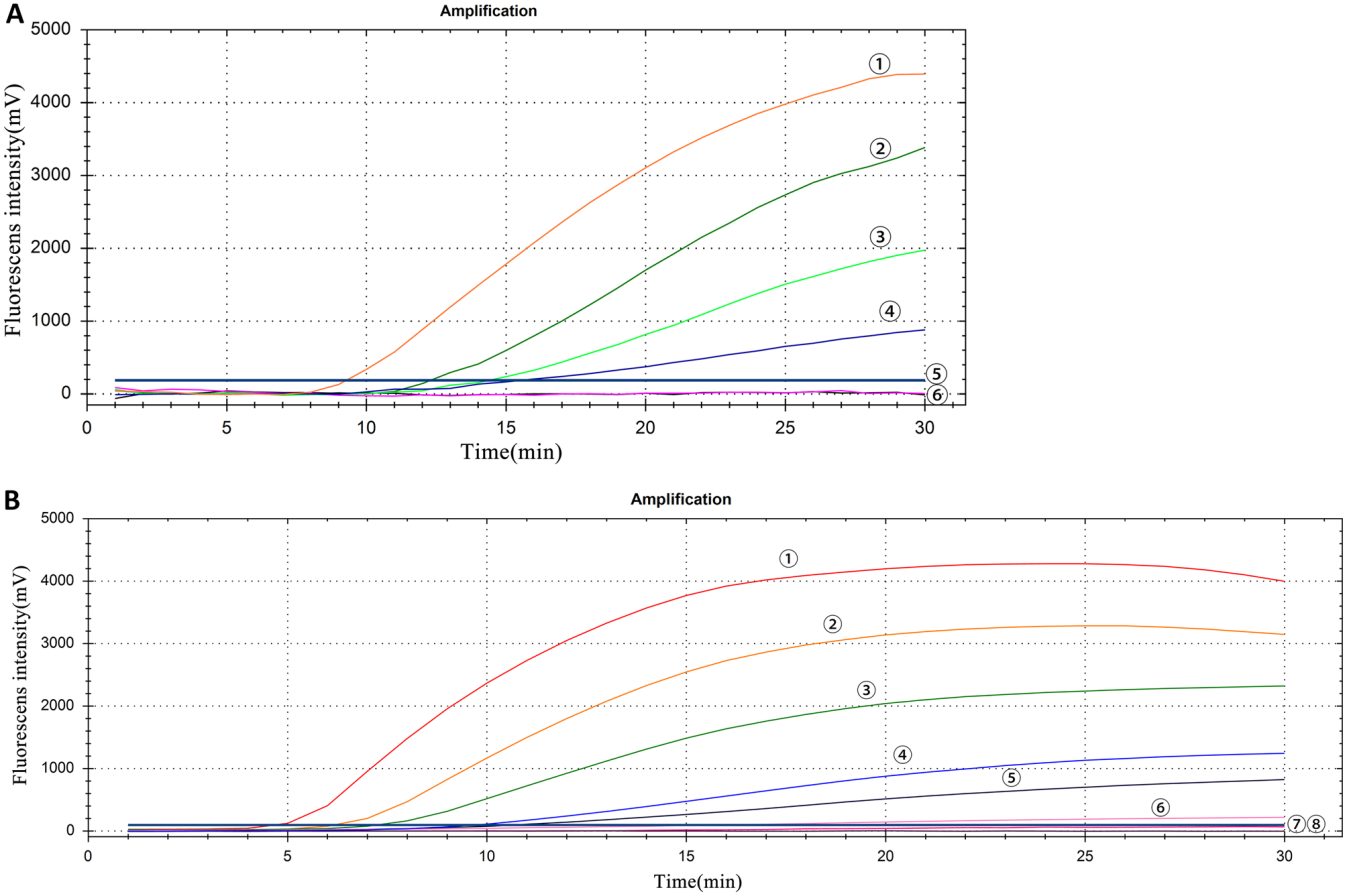
RT-RAA sensitivity test results. (**A**) GETV viral RNA as the standard, ①–⑤: 2 × 10^4^–2 × 10^0^ TCID_50_/reaction in turn; ⑥: negative control. (**B**) GETV plasmid transcript as standard, ①–⑦: 8 × 10^5^–8 × 10^−1^ copies/reaction in turn; ⑧: negative control.

Only GETV produced fluorescence signals, while other viruses and nuclease free water did not produce fluorescence signals (Fig. 3). The sensitivity of this method was assessed using gradient dilution plasmid transcripts of the 8 × 10^5^–8 × 10^−1^ copies/reaction and GETV viral RNA of the 2 × 10^0^–2 × 10^4^ TCID^50^/reaction, with each dilution measured eight times to assess the reproducibility of the method (Table 1). The minimum detection limitation for this method is 8 copies/reaction plasmid transcripts and 20 TCID_50_/reaction viral RNA. The higher the template concentration is, the greater the fluorescence value and the earlier the peak time is (Fig. 4).

**Table 1:** Sensitivity of duplex RT-RAA assays using cultured GETV RNA and plasmid.

Serially diluted GETV culture medium (TCID_50_/reaction)	Serially diluted GETV plasmid (copies/reaction)	No. replicates tested	No. detection	Detection rate (%)
–	8 × 10^5^	8	8	100
–	8 × 10^4^	8	8	100
2 × 10^4^	8 × 10^3^	8	8	100
2 × 10^3^	8 × 10^2^	8	8	100
2 × 10^2^	8 × 10^1^	8	8	100
2 × 10^1^	8 × 10^0^	8	8	100
2 × 10^0^	8 × 10^−1^	8	0	0

### Detection of GETV in clinical samples

Among the 118 clinical samples, 19 positive samples were detected by RT-RAA and the same results were showed by real-time RT-qPCR. The coincidence rate of the two detection methods is 100% (Table 2).

**Table 2:** Comparison of GETV detection examined RT-RAA and SYBR Green I RT-PCR.

Tissue	No. of samples	No. SYBR Green I RT-qPCR	No. RT-RAA
Kidney	12	1	1
Spleen	30	9	9
Intestine	8	5	5
Lungs	15	2	2
Liver	15	1	1
Brain	11	1	1

## Discussion

RAA is a new nucleic acid detection technology, which has a unique set of primer and probe design principles. The length of primers should be 30–35 bp, and the amplified fragment should be no less than 70 bp and no more than 500 bp, preferably controlled at 100–200 bp. The probe length was 46–52 bp, and the restriction site was at least 30 bp away from 5′ and at least 15 bp away from 3′. Compared with the primers and probes for RT-qPCR, the use of longer primers and probes for RT-RAA also improved the specificity of the method. In the process of primer and probe design, the conserved region of GETV non-structural protein gene NSP1 gene sequence was selected in this study. The GETV genome is highly conserved, with 93–100% homology among the sequences of isolated GETV strains. Because the non-structural protein gene sequence is highly conserved in the genome, but contains enough genetic variation to distinguish it from other alphavirus, the non-structural protein gene NSP1 is often used as the target gene in GETV nucleic acid detection methods^23–25^.

As an emerging nucleic acid detection technology, RAA has the characteristics of shorter time consumption, higher sensitivity and stronger specificity compared with traditional detection methods. Respiratory syncytial virus RT-RAA method established by Chen et al.^26^ has a sensitivity of 35 copies/reaction, and results can be obtained within 30 min, while RT-qPCR requires 2–3 h. Conventional RT-PCR takes much longer. In this study, a fluorescent RT-RAA method for rapid detection of GETV was established. The fluorescence signal could be detected in 10 min after rapid amplification at 39 °C, and the results could be obtained within 30 min. The detection method of GETV TaqMan RT-qPCR established by Zhu et al.^23^ requires 3–4 h to obtain the detection results. The detection limit of this method for GETV plasmid was 8 copies/reaction, and the detection limit for GETV viral RNA was 20 TCID50/reaction, which was similar to the sensitivity of the GETV RT-LAMP detection method established by Liu et al.^27^. The sensitivity was slightly higher than that of GETV TaqMan RT-qPCR established by Shi^25^, which was 100 times that of ordinary RT-PCR. The GETV RT-RAA method established in this study has no cross-reaction to other viruses. In the detection of clinical samples, the coincidence rate of RT-RAA and SYBR Green I RT-qPCR was 100%, with high accuracy. In this study, a rapid, specific and sensitive detection method for GETV was established, which can be applied to the clinical diagnosis of GETV.

GETV RT-RAA was used to detect 118 clinical samples, and 19 samples were positive for GETV. All the 19 positive samples were from swines with mild diarrhea in the same swine farm, indicating that GETV can exist in the swine farm for a long time and threaten the health of the swine population. The GETV fluorescent RT-RAA detection method established in this study has high specificity and sensitivity, and can be used for the monitoring of GETV in swines to timely understand the health status of swines. In the analysis of the collection time of positive samples, we found that the positive rate of GETV was higher in summer and autumn, which are also the prevalent seasons of mosquitoes. It is speculated that mosquitoes may accelerate the transmission of GETV in swines. Existing studies have also proved that mosquitoes in swine farms can spread GETV among swines. Therefore, in the prevention and control of GETV, we need to pay attention to the prevention and control of mosquitoes, carry out preventive control of mosquitoes before the outbreak, and cut off the risk of mosquito transmission of GETV.

The GETV rapid detection of fluorescent RT-RAA method established in this study has the characteristics of high sensitivity, strong specificity and short diagnosis time, and can be used for early diagnosis and long-term monitoring of GETV. For the prevention and control of GETV, it is necessary to increase the research and development of antiviral drugs and vaccines while achieving early diagnosis. Abdelnabi^28^ summarized the drug action targets and drug screening tools of anti-arthritis arthrovirus, and successfully screened anti-chikungunya virus drugs. In Japan, Izumida^29,30^ constructed a weak strain of GETV KB/VT as a candidate strain for live vaccine of porcine GETV virus, and a commercial porcine GETV virus vaccine has been developed. However, in China, there is little research and development of GETV vaccine. Fu et al.^31^ isolated a GETV JS18 strain, and used this strain to develop inactivated vaccine and evaluate the immune efficacy in swines. There is a lack of research on live vaccines, genetically engineered vaccines, recombinant vaccines and other vaccines. It is believed that with the increasing attention to GETV in China, our research on it will be more in-depth and more conducive to the effective prevention and control of GETV.

## Methods

### Virus and clinical samples

GETV SC201807 was isolated and identified by the Animal Biotechnology Center of Sichuan Agricultural University^32^. A total of 118 clinical samples were collected from kidney, spleen and intestines of pigs that died of diarrhea or abortion in Sichuan from September 2020 to May 2021.

### Primer and probe design

The non-structural gene NSP1 sequences of GETV from different regions were downloaded from GenBank (the gene homology in the conserved region was 99.0–100%), and the conserved region was analyzed using DNAMAN software, and a pair of RT-PCR primers and three pairs of RT-RAA primers and probes were designed (Supplementary Table 1).

### RNA extraction

RNA was extracted from the virus and samples using Cofitt^®^Total RNA Reagent (Hong Kong Kefit Co., Ltd), and stored at − 80 °C.

### Production of a standard control

RT-PCR primers were used to amplify GETV virus. After gel recovery, the amplified product was linked to Peasy-Blunt Zero Cloning Kit, and transformed into *Escherichia coli* DH5α capable cell culture. After that, plasmid was extracted and the concentration was determined. After plasmid digestion and linearization, the standard plasmid transcript was transcribed in vitro using the MMessage MMachine™ T7 Transcription Kit (Thermo Fisher Scientific). After purification, the standard plasmid transcript was stored at − 80 °C. The TCID_50_ of GETV SC201807 strain was 10^6.85^/0.1 mL, and the gradient dilution was 10^6^–10^2^ TCID_50_/0.1 mL.

### Real-time RT-RAA assay and primer selection

Fluorescent RT-RAA nucleic acid amplification kit (Jiangsu Qitian Gene Biotechnology Co., Ltd.) was used, including reverse transcription and DNA amplification enzymes. The total volume of RT-RAA reaction system is 50μL (Table 3). After the system was configured, it was immediately transferred to a fluorescence quantitative PCR instrument preheated to 39 °C. The denaturation temperature, annealing temperature and extension temperature were all set at 39 °C, and each cycle was 1 min and 30 cycles were set. Nuclease–free water was used as negative control.

**Table 3:** RT-RAA reaction system.

Reagent	Concentration	Content/μL
Buffer	–	25.0
Forward primer	10 μM	2.1
Reverse primer	10 μM	2.1
Probe	10 μM	0.6
ddH2O and RNase inhibitor	–	15.2
Template	–	2.0
Reaction starter	–	3.0

Three forward primers and three reverse primers of RT-RAA were combined. From 1 to 9, they were F1/R1, F1/R2, F1/R3, F2/R1, F2/R2, F2/R3, F3/R1, F3/R2, F3/R3, F3/R3. And the combined primers were used for RT-PCR detection. The amplified strips were connected with pEASY-Blunt Zero Cloning Kit after glue recovery, and the ligation products were sent to Sangon Bioengineering (Shanghai) Co., Ltd for sequencing. Whether the alignment and sequencing results are GETV sequences. The combined 9 pairs of primers were used to detect GETV viral RNA, and the primers were screened by fluorescence value and peak time.

### Sensitivity and specifcity of real-time RT-RAA

The sensitivity of the method was assessed using 8 × 10^5^–8 × 10^−1^ copies/reaction transcripts and 2 × 10^0^–2 × 10^4^ TCID_50_/reaction RNA. The specificity of the method was tested by Porcine reproductive and respiratory syndrome (PRRSV), Japanese encephalitis virus (JEV), Atypical porcine pestivirus (APPV), Seneca valley virus (SVV) and Classical swine fever virus (CSFV).

### Clinical specimen analysis

The RNA of total 118 clinical samples were collected for fluorescence RT-RAA detection. At the same Time, PrimeScript™ RT Master Mix (Perfect Real Time) (Takara Biomedical Technology (Beijing) Co., Ltd.) was used for reverse transcription to obtain cDNA. cDNA was detected by RT-qPCR using TB Green^®^ Premix Ex Taq™ (Tli RNaseH Plus) (Takara Biomedical Technology (Beijing) Co., Ltd.). RT-qPCR system and procedure are shown in Table 4. And sequences of primers for RT-qPCR in Supplementary Table 2.

**Table 4:** SYBR Green I RT-qPCR system and procedure.

RT-qPCR system		RT-qPCR procedure		
Reagent	Content/μL	Temperature (°C)	Time	Cycle
TB Green Premix Ex Taq	12.5	95	5 min	1
Forward primer	1.0	95	30 s	35
Reverse primer	1.0	56	30 s	
ddH_2_O	9.5	72	30 s	
Template	1.0	42	7 min	1

## Supplementary Information

Supplementary Information.
