# Supplementary material for: Development of a reverse transcription recombinase-aided amplification assay for detection of Getah virus
Source: Sci Rep. 2021 Oct 8;11:20060. doi: 10.1038/s41598-021-99734-7 (PMC8501081; doi:10.1038/s41598-021-99734-7)
Supplement: Supplementary file 1 — Supplementary Information. [file 41598_2021_99734_MOESM1_ESM.docx]

**Development of a Reverse transcription recombinase-aided amplification assay for detection of Getah virus**

**NIE Mincai^1^, DENG Huidan^1^, ZHOU Yuanche****ng^3^, SUN Xiangang^1^, HUANG Yao^1^, ZHU Ling^1,2^****^*^, XU Zhiwen^1,2^**

Supplementary Table 1. Sequences of Primers and Probe for Assays for GETV

| Primer/Probe | Sequence (5'-3') | Length（bp） | Tm（℃） |
| --- | --- | --- | --- |
| RT-RAA-F1 | ATTGAGCAAGAGGTTCCAACAGGCGTCACCATC | 33 | 67 |
| RT-RAA-F2 | TGATGTCTGACCACACCTACCACTGCATCTG | 31 | 65 |
| RT-RAA-F3 | ACCCAGAGAGGCTGGCGAATTACGCTCGAAAG | 32 | 68.6 |
| RT-RAA-R1 | TCAGCCCTAGTGCGGCACGTCTCGTCAGTRTG | 32 | 70.9 |
| RT-RAA-R2 | TCGGGGATTCCAAGTCTGGAGTGGCCATGACG | 32 | 70.3 |
| RT-RAA-R3 | CACGTCTCGTCAGTATGCAGGCAAAAAGTCG | 31 | 64.7 |
| RT-RAA-P | CTTGTAGGTCCGTTATCTTTCCGGACACAT/I6FAMDT/  IDSP/IBHQ1DT/TGTCTAGCACAGTC | 46 | 66.8 |
| RT-PCR-F4 | CCGAATGACCATGCTAACGCT | 21 | 61.1 |
| RT-PCR-R4 | TCCAATCCAATACGCCGTCCT | 21 | 61.6 |

Supplementary Table 2: Sequences of Primers for RT-qPCR

| Primer | Sequence (5'-3') | Length（bp） | Tm（℃） |
| --- | --- | --- | --- |
| cap-F | CTTGACGGTAAGGTCACGGG | 20 | 60.39 |
| cap-R | GTAAGCTTCGCTAGGTCGGG | 20 | 60.25 |
